# Dietary Iron Intake in Excess of Requirements Impairs Intestinal Copper Absorption in Sprague Dawley Rat Dams, Causing Copper Deficiency in Suckling Pups

**DOI:** 10.3390/biomedicines9040338

**Published:** 2021-03-27

**Authors:** Jennifer K. Lee, Jung-Heun Ha, James F. Collins

**Affiliations:** 1 Food Science and Human Nutrition Department, University of Florida, Gainesville, FL 32611, USA; leejennifer@ufl.edu; 2 Research Center for Industrialization of Natural Neutralization, Dankook University, Cheonan 31116, Korea; ha@dankook.ac.kr; 3 Department of Food Science and Nutrition, Dankook University, Cheonan 31116, Korea

**Keywords:** iron supplementation, ceruloplasmin, pregnancy, lactation, SOD1

## Abstract

Physiologically relevant iron-copper interactions have been frequently documented. For example, excess enteral iron inhibits copper absorption in laboratory rodents and humans. Whether this also occurs during pregnancy and lactation, when iron supplementation is frequently recommended, is, however, unknown. Here, the hypothesis that high dietary iron will perturb copper homeostasis in pregnant and lactating dams and their pups was tested. We utilized a rat model of iron-deficiency/iron supplementation during pregnancy and lactation to assess this possibility. Rat dams were fed low-iron diets early in pregnancy, and then switched to one of 5 diets with normal (1×) to high iron (20×) until pups were 14 days old. Subsequently, copper and iron homeostasis, and intestinal copper absorption (by oral, intragastric gavage with ^64^Cu), were assessed. Copper depletion/deficiency occurred in the dams and pups as dietary iron increased, as evidenced by decrements in plasma ceruloplasmin (Cp) and superoxide dismutase 1 (SOD1) activity, depletion of hepatic copper, and liver iron loading. Intestinal copper transport and tissue ^64^Cu accumulation were lower in dams consuming excess iron, and tissue ^64^Cu was also low in suckling pups. In some cases, physiological disturbances were noted when dietary iron was only ~3-fold in excess, while for others, effects were observed when dietary iron was 10–20-fold in excess. Excess enteral iron thus antagonizes the absorption of dietary copper, causing copper depletion in dams and their suckling pups. Low milk copper is a likely explanation for copper depletion in the pups, but experimental proof of this awaits future experimentation.

## 1. Introduction

Iron deficiency (ID) in humans is common in developed countries, most frequently occurring due to blood loss, inappropriately low intestinal iron absorption (given body iron status) or during physiological states of increased demand [1,2,3]. Those at greatest risk for developing ID thus include premature babies, young children and adolescents [4], pregnant women, women of child-bearing age with menorrhagia and the elderly. ID has also been associated with obesity, vegetarianism, chronic use of acid blockers (used to treat acid reflux), gastric bypass surgery for the treatment of morbid obesity, and short-bowel syndrome and other malabsorptive disorders (e.g., Celiac disease). Moreover, chronic inflammatory conditions also frequently result in ID (and the anemia of inflammation), due to proinflammatory cytokine-induced upregulation of the iron-regulatory hormone hepcidin (which functions, in part, to suppress intestinal iron absorption). To prevent development of iron deficiency, those at increased risk are advised to consume foods rich in bioavailable iron (most notably, meat products containing heme iron) and vitamin C (which enhances intestinal iron absorption), but for many, iron supplementation is likely to also be required.

Iron depletion during pregnancy has been frequently observed, as many women have insufficient iron stores to meet the increased demand associated with fetal growth and development and expansion of the maternal blood supply. Moreover, many pregnant women do not achieve the recommended daily iron intake of 27 mg/day (Dietary Reference Intakes for Iron), which increases risk for ID, anemia, and negative pregnancy outcomes. Iron supplementation is thus often recommended during pregnancy [5,6,7,8]. For example, the World Health Organization recommends daily oral supplementation with 30–60 mg of elemental iron for pregnant women to prevent maternal anemia, puerperal sepsis, low birth weight, and preterm birth (WHO Guidelines for Iron Supplementation During Pregnancy). The WHO further recommends that any woman diagnosed with anemia during pregnancy increase daily iron intake to 120 mg/day until the anemia resolves. Not surprisingly, iron supplementation seems to be most beneficial for women with ID or anemia, while supplementation of iron replete women would be predicted to be without positive effect. Indeed, it has been recommended that iron supplementation of pregnant women be implemented with caution, since too much iron can also lead to negative pregnancy outcomes [9]. Furthermore, a recent meta-analysis concluded that results of iron supplementation programs showed a reduced risk of maternal ID and anemia, but positive influences on other maternal and infant outcomes were less clear due to heterogenous, individualized responses to increased oral iron intake [10]. Therefore, in sum, iron supplementation during pregnancy is widely recommended but should nonetheless be approached with caution.

It has been noted previously that high iron intakes may antagonize copper homeostasis [11,12]. This may relate to impaired copper absorption when iron intakes are high, as documented in infants and adults [11]. Additional recent investigations supported these postulates, as increases in dietary iron resulted in copper deficiency-related pathological outcomes in adolescent rats [13,14] and mice [15]. Copper supplementation of mice consuming a high-iron diet prevented the development of these pathologies, proving that they related specifically to copper depletion [16]. Collectively, these investigations suggested that high iron consumption increases the dietary requirement for copper, supporting the previous assertion that iron supplements should contain extra copper [11]. Moreover, given that many individuals in the U.S. may have marginal copper status [11,17], and since refined grain products are fortified with iron and iron is found in most daily vitamin/mineral supplements, copper depletion from excess iron consumption is of concern. This situation could be most detrimental during pregnancy, when iron demand increases and iron supplementation is more likely to be recommended, as copper deficiency disrupts normal fetal growth and development [18,19]. This background then forms the basis for the current investigation, which was designed to test the hypothesis that increasing dietary iron above requirements will cause copper depletion during pregnancy. We thus utilized a rat model of ID/iron supplementation in pregnancy and lactation to assess the impact of excess dietary iron on copper homeostasis of the dam and the suckling pups.

## 2. Materials and Methods

### 2.1. Experimental Design and Dietary Manipulations

**Figure 1:**
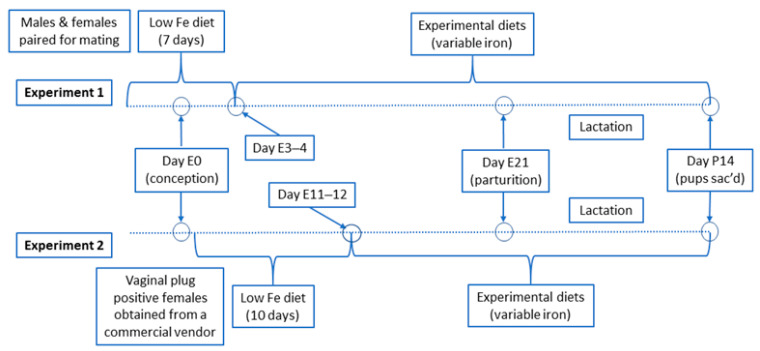
Experimental Design Schematic. Days with an ‘E’ designation indicate embryonic day number (i.e., after conception); ‘P’ indicates postnatal day number (i.e., after birth). The days listed in the diagram are our best estimates, as the exact time/day of conception for each dam is not precisely known. Additionally, note that the litter sizes in experiment 2 were equalized to 8 pups each (to eliminate confounding variables related to the number of suckling pups).

All animal studies were approved by the University of Florida Institutional Animal Care and Use Committee (Study #202001829; approved 10/30/2020). Two rat pregnancy studies were performed: (1) an initial (pilot) proof-of-principle study; and (2) a follow up study with an optimized experimental design. In experiment one, 8-week-old male and female rats were obtained from a commercial vendor (Envigo; Hayward, CA, USA) and paired for mating upon arrival. In experiment two, nine-week-old, timed pregnant (i.e., vaginal plug positive) Sprague Dawley rats were obtained from Envigo. All rats were initially fed a standard chow diet. Rats were then switched to an iron-deficient diet (2–6 ppm Fe; TD.120105; Envigo) for either 7 days beginning when rats were paired for mating (experiment 1) or for 10 days beginning in early pregnancy (experiment 2). Thereafter, pregnant female rats were randomized and fed one of five experimental AIN-93G-based diets (Dyets Inc.; Bethlehem, PA, USA) with variable iron levels (adequate to high) and marginal copper content, throughout the remainder of pregnancy and during lactation (until the pups were 13–15 days of age) (the experimental design is shown in Figure 1).

The target iron concentrations in the 5 experimental diets were (in ppm) 80, 240, 400, 800, and 1600. Although the AIN-93G diet has 50 ppm iron, we have routinely used iron at 80 ppm for pregnancy and lactation studies (based upon personal experience and in consultation with a collaborator and experienced iron/copper researcher, Joseph Prohaska (Prof. Emeritus, University of MN, Duluth)). We thus considered the 80-ppm diet as the control (i.e., normal) iron (1×) diet. During the anemia of pregnancy in humans, iron supplementation up to several times above the RDA is frequently recommended (e.g., 120 mg/day in some cases, which is ~4.5 times the RDA for pregnant women). Additionally, when one considers iron present in a natural, balanced diet (including highly bioavailable heme iron), consumption of iron fortified foods (e.g., refined grain products) and iron contained in daily multi-vitamin/mineral supplements, it is easy to imagine a situation where iron intake could be up to 10 times above recommendations for pregnant women. We thus considered the 240-ppm iron (3×) diet and 400-ppm iron (5×) diet to be well within the range of iron intake for pregnant women (with supplementation, which is likely), and the 800-ppm iron (10×) diet to be the upper range of possible intakes. The 1600-ppm iron (20×) diet, which clearly contained supra-physiological iron levels, was used as a comparator of a maximal response. The composition of the diets is shown in Table 1 and Table 2.

Our intent was to model the anemia of pregnancy in humans, when iron supplementation (up to several times above the Recommended Dietary Allowance (RDA), in some cases) is frequently recommended [7,8]. Thus, experimental rats were first subjected to dietary iron restriction to induce iron deficiency and/or anemia, and then fed diets with supplemental iron (to mimic iron supplementation in humans). Moreover, since many Americans may have marginal copper intakes [11,17], we decreased the copper content of the experimental diets from 6–7 ppm, which is standard in the AIN-93G diet, to a target concentration of 2–3 ppm. All diets were essentially isocaloric (3760 kcal/kg), with very slight (insignificant) decreases in the 5× (0.03%), 10× (0.08%) and 20× (0.16%) diets (since carbonyl iron replaced small amounts of sucrose). For all experiments, rats were housed in standard shoebox cages with ad libitum access to diet and purified water. Body weight of the rats and food intake were measured every 3–4 days. For experiment two, the number of pups was adjusted to 8 in each litter (by euthanizing the remaining pups from larger litters). This was done to ensure that all pups had equal chances of obtaining adequate milk from the dam (since we intended to do a ^64^Cu absorption study in the dams and assess ^64^Cu delivery to the pups through the milk). Subsequently, dams and pups were killed (when pups were ~14 days of age) by thoracotomy after CO_2_ narcosis. Blood was collected by cardiac puncture, and various organs were removed and weighed. Tissue and blood samples were stored at −80 °C until experimental analyses were performed.

### 2.2. Quantification of Hematological Parameters, Serum and Tissue Nonheme Fe Levels, and Copper Biomarker Assays

Blood hemoglobin (Hb) and hematocrit (Hct) levels were determined using standard assays, as previously described [15]. Serum and hepatic nonheme iron concentrations and total iron-binding capacity (TIBC) were determined using routine colorimetric assays [15,20]. Transferrin saturation (TSAT) was calculated as ((serum nonheme iron concentration/TIBC) × 100). Identification of copper-related biomarkers in mammals has historically been a challenge [21,22], but a few reasonable candidates exist, including serum Cp activity [23], erythrocyte copper, zinc superoxide dismutase 1 (SOD1) activity [24], and serum peptidyl-glycine α-amidating monooxygenase (PAM) activity [25]. Thus, a *para*-phenylenediamine (*p*PD) oxidation assay [26] was used to quantify serum Cp activity, as previously described [14]. Erythrocyte SOD1 activity was measured using the Superoxide Dismutase Activity Assay kit (ab65354; Abcam; Cambridge, MA, USA). Serum PAM activity was measured using a rat ELISA Kit (MBS9325946; MyBioSource; San Diego, CA, USA), following the manufacturers’ instructions.

### 2.3. Quantification of Iron and Copper Concentrations in Experimental Diets and Copper in Liver

Ten randomly selected pellets from each diet were ground with an acid-washed mortar and pestle. Ground diet samples were digested with HNO_3_/H_2_O_2_ on a hot block. Digested samples were filtered (0.45 μm) and analyzed by ICP-MS (NexIon 300; Perkin-Elmer Corp.; Waltham, MA, USA). Iron and copper concentrations in diets were normalized by premeasured weights. Dried rodent livers, for which wet weights were pre-determined, were transferred to perfluorooxyalkane (PFA) digestion vessels. Two mL 50% Optima HNO_3_ and 0.2 mL 30% ACS grade H_2_O_2_ (Fisher Chemicals; Woodlawn, NJ, USA) were added to the livers. A method blank and Standard Reference Material (SRM) 1577c Bovine Liver (NIST; Gaithersburg, MD, USA) were prepared along with the samples. The PFA vessels were sealed and placed into a microwave digestion oven (CEM; Matthews, NC, USA). The digestion program was as follows: (a) 1600 W at 80% power, temperature ramp to 200 °C within 15 min, (b) hold at 200 °C for 15 min, and (c) cool down 15 min. After complete cooling, sample digests were quantitatively transferred to polypropylene tubes and diluted with ≥18.2 MΩ∙cm water (Millipore Sigma; Burlington, MA, USA) up to 15 mL. Metal analysis was accomplished at the University of Florida Analytical Toxicology Core Laboratory with an Agilent 7900 ICP-MS (Agilent Technologies; Santa Clara, CA, USA) equipped with in-line internal standard addition. The instrument was operated in He gas mode, which minimized polyatomic interferences. Calibration dilutions ranged from 0.1 to 10,000 ng/mL; resulting calibration regressions were linear with *r*^2^ ≥ 0.999. Measured Cu in SRM 1577c came to within ≈10% of the published value, and 20% was determined as the cut-off limit for accuracy.

### 2.4. Intestinal Copper Absorption Study

^64^Cu absorption and tissue distribution, and delivery to the suckling pups was assessed as part of experiment 2. When pups were 13–14 days old, dams were fasted overnight (~16 h) with free access to purified water, and then administered 4 mCi ^64^CuCl_2_ (4314 mCi/mL; Washington University; St. Louis, MO, USA) diluted into PBS containing 0.1 N HCl by oral, intragastric gavage. Immediately thereafter, rats were given unrestricted access to their respective experimental diets (to stimulate digestion/absorption), and then killed 48 h later. Subsequently, the amount of radioactivity in each carcass (after the GI tract was removed) was quantified at a fixed distance using a Ram DA Gamma Counter with a PM-11 tube (Rotem Industries; Arava, Israel). This method was utilized since we do not have access to a gamma counter that can accommodate a rat carcass. The 48-h time point was chosen for technical and experimental reasons: first, given the timing of the experiments, a shorter time point was not technically feasible; and second, 48 h should allow for all mucosal copper to be absorbed, including any temporarily stored within intestinal epithelial cells (e.g., in metallothionein). There is precedence for a slower component of metal ion absorption reflecting release from storage, as for example, with iron. Most enteral iron is absorbed quite rapidly (within a few hours), but there is another slower component that reflects iron release from storage in enterocytes (in ferritin), that is thought to occur over 12–20 h [27]. Furthermore, although biliary excretion could also have influenced whole-body ^64^Cu levels, copper loss in bile would be expected to be extremely low (since dams were fed a moderate copper diet and were copper deficient). Total counts in each carcass then logically reflect the magnitude of copper absorption. Radioactivity in blood and various organs was measured using a WIZARD^2^ automatic gamma counter (Perkin-Elmer Corp.; Waltham, MA, USA). All radioactivity counts were corrected for the short half-life of ^64^Cu (i.e., 12.7 h).

### 2.5. Statistical Analysis

Results are presented as box-and-whisker plots displaying the minimum, the lower (25th percentile), the median (50th percentile), the upper (75th percentile), and the maximum ranked sample. Data were analyzed by one- or two-way ANOVA using GraphPad Prism (v9.0). When significant differences were noted by one-way ANOVA, or when a significant interaction was noted by two-way ANOVA, Tukey’s post hoc test was run to identify differences between individual groups. *p* < 0.05 was considered statistically significant. Pearson product-moment correlation coefficient (*r*) was calculated in cases where additional interpretations were required.

## 3. Results

### 3.1. Iron and Copper Content of Experimental Diets

Dietary iron and copper concentrations were measured by ICP-MS. Diets contained iron and copper close to target values (described above), as follows (in mg/kg (or ppm)): iron content—88 (1×), 235 (3×), 422 (5×), 886 (10×), and 1598 (20×); copper content varied between 2.44 and 3.01 (Table 2).

#### 3.1.1. Experiment 1: High-Iron Intake by Rat Dams Suppresses Serum pPD Oxidase Activity and Increases Hepatic Nonheme Iron in Suckling Pups

The first experiment was a proof-of-principle study designed to test the hypothesis that high-iron consumption by previously iron deficient pregnant/lactating rat dams would cause copper deficiency in suckling pups. We did not assess iron status in dams after dietary iron restriction since handling of pregnant rats is likely to lead to negative pregnancy outcomes (e.g., miscarriage). However, we feel that it is quite likely that they were indeed iron deficient (and possibly anemic), based upon personal experience working with SD rats and a supportive publication [28]. Body weight and average daily calorie intake of the dams were not significantly different between experimental groups (data not shown). Moreover, there were no significant differences between dams in any of the experimental groups with respect to serum Hb levels, serum nonheme iron content, TSAT or serum *p*PD oxidase activity (Table 3). TIBC was significantly lower in the 3×, 5×, 10× and 20× groups (Table 3).

Organ weights of the dams also did not differ significantly among the 5 dietary groups (Table 4). Organ weights of the pups showed only minor variation between dietary groups, but nonetheless, significant main effects were noted (by 2-way ANOVA) (Table 4). The biological significance of these exceedingly small differences is questionable at best.

**Figure 2:**
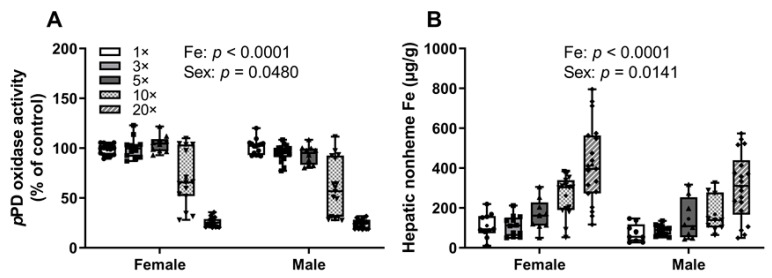
High-iron consumption by previously iron-deficient rat dams decreases serum *p*PD oxidase activity and increases hepatic nonheme iron content in their offspring. Serum *p*PD oxidase activity (**A**) and hepatic nonheme iron content (**B**) of 13–15-day-old pups are shown. Pups were suckling rat dams fed diets varying only in iron concentration for ~5 weeks, starting around the time of conception. Results were analyzed by two-way ANOVA; no significant 2-way interactions were noted. Significant main effects are noted in each panel. All data points are shown, and the mean values are indicated by “+” signs.

Serum Hb levels varied slightly among groups in male and female pups (mainly between 8 and 9 g/dL) (data not shown), but no consistent patterns were noted even though significant iron (*p* = 0.014) and sex (*p* = 0.0289) main effects were documented. Serum *p*PD oxidase (i.e., Cp) activity was decreased in the 10× (~35%) and 20× (~75%) groups in both sexes of pups (as compared to the other groups) (Figure 2A). This decrease in serum Cp activity most likely reflects copper depletion, as Cp is an established biomarker of moderate to severe copper deficiency [29,30]. Furthermore, hepatic nonheme iron concentrations progressively increased in both male and female pups as the iron content of the dam’s diet increased (Figure 2B). This observation is consistent with the concept that serum Cp activity is required for iron release from hepatocytes [31,32,33]. In sum, progressive increases in dietary iron consumed by rat dams caused copper deficiency in suckling pups. Although there were no decrements in serum Cp activity observed in the dams, it seems likely that they were indeed copper deficient. Cp activity may not change in less severe copper deficiencies; thus, in experiment 2, as part of the optimized experimental design, we used a wider variety, and possibly more sensitive biomarkers of copper status, and hepatic copper levels were also quantified.

#### 3.1.2. Experiment 2: Optimized Experimental Design

Given outcomes and limitations revealed in the first experiment, in the second experiment, we altered the experimental design by starting the iron deprivation regimen early in pregnancy and extended it to 10 days. Thus, in experiment 1, after 7 days of dietary iron deprivation during the mating period, females (some pregnant) were switched to the experimental diets around the 3rd or 4th day of gestation. In experiment 2, after 10 days of dietary iron deprivation starting 1–2 days after conception, dams were switched to experimental diets at around day 11–12 of gestation (Figure 1). The dams were thus placed on the experimental (i.e., variable iron) diets several days later in pregnancy (than in experiment 1). Litter sizes were also equalized to 8 pups/dam, to eliminate confounding variability associated with different litter sizes. We also utilized additional assays to detect copper deficiency in the dams and suckling pups.

#### 3.1.3. Experiment 2: High-Iron Consumption Perturbs Iron and Copper Homeostasis in Dams

Body weight and average daily calorie intake of the dams were not significantly different between groups (as in experiment 1) (data not shown). Kidney, spleen, and heart weights were not different among the different dietary groups (Table 5). Hepatomegaly was noted in the dams as liver weight (as a % of BW) significantly increased as the dietary iron concentration increased (Table 5).

**Figure 3:**
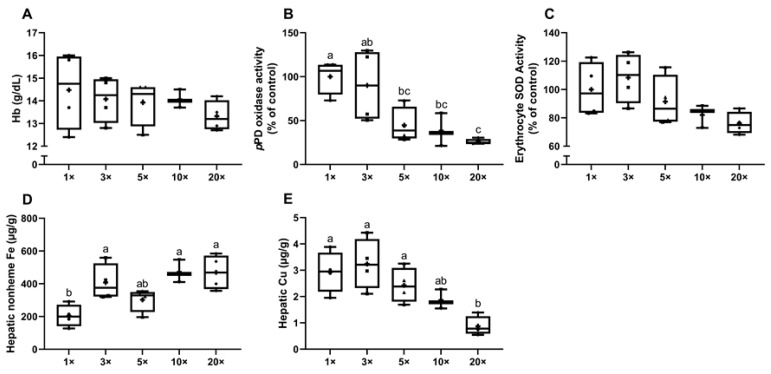
Consumption of a high-iron diet by previously iron-deficient rat dams perturbs iron and copper homeostasis. Blood hemoglobin (Hb) levels (**A**), serum *p*PD oxidase activity (**B**), erythrocyte SOD1 activity (**C**), hepatic nonheme iron content (**D**), and hepatic copper concentrations (**E**) were assessed in dams fed diets varying in iron concentration for ~3.5 weeks during pregnancy and lactation. One-way ANOVA analysis results are as follows: *p*PD oxidase activity (**B**), *p* = 0.0023; hepatic nonheme Fe content (**D**), *p* = 0.0038; hepatic Cu content (**E**), *p* = 0.0022. When statistically significant differences were noted by one-way ANOVA, Tukey’s post hoc test was utilized to make comparisons between individual groups. Values without common superscript letters are statistically different from one another (*p* < 0.05). (**B**,**D**,**E**). All data points are shown, and the mean values are indicated by “+” signs.

Hemoglobin levels in dams were not significantly different, but trended lower in the 20× group (Figure 3A). Since no ‘gold standard’ copper-related biomarker has been identified [22,34], three cuproenzyme activities were measured to determine copper status, including the activities of serum Cp and PAM, and erythrocyte SOD1 [21]. Serum *p*PD oxidase activity was reduced ~55% in the 5× group, ~61% in the 10× group and ~74% in the 20× group (all in comparison to the 1× group) (Figure 3B). Although significance was not noted, erythrocyte SOD1 activity (percent inhibition) showed a decreasing trend in the 10× (~18%) and 20× (~23%) groups (Figure 3C). No changes in serum PAM activity were documented between groups (data not shown). Hepatic nonheme iron content increased ~2 fold in the 3× group and ~2.3-fold in the 10× and 20× groups (Figure 3D). Additionally, hepatic copper content trended lower in the 10× group (but did not reach statistical significance) and was significantly decreased in the 20× group (>3-fold lower than the 1× group), as compared to all others (Figure 3E). Thus, progressive increases in iron consumption caused copper deficiency in the dams, as evidenced by reductions in serum Cp activity and decreased liver copper content. It is likely that reductions in hepatic copper resulted in less metalation of *apo*-Cp in the liver, thus decreasing active (*holo*-) Cp enzyme in the blood (note that *apo*-Co is quite unstable and has a short half-life in serum) [35]. Also, increased liver iron loading probably reflected impaired Cp-dependent iron release from hepatocytes.

#### 3.1.4. Experiment 2: High-Iron Consumption by Rat Dams Alters Final Body Weights, Serum pPD Oxidase and Erythrocyte SOD1 Activities and Hepatic Copper Content in Suckling Pups

Liver, kidney, and spleen weights in the pups (as a % of BW) were not different among the groups (Table 6). Heart weights varied slightly across experimental groups, but no meaningful trend was noted (Table 6).

**Figure 4:**
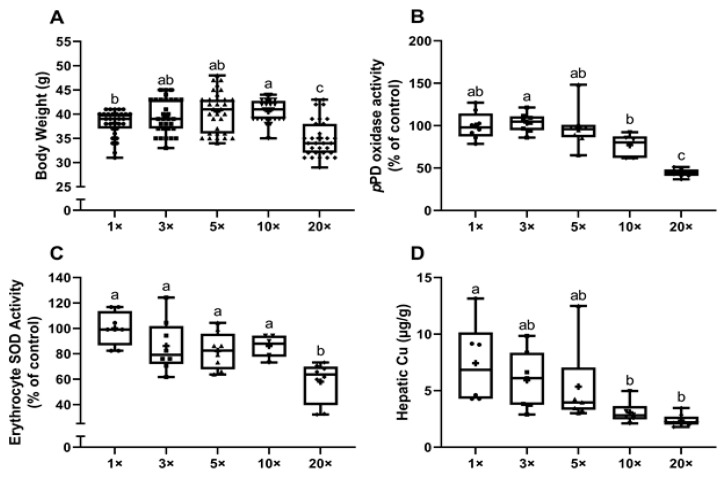
High-iron consumption by rat dams impairs growth, reduces serum *p*PD oxidase and erythrocyte SOD1 activities, and decreases hepatic copper content in suckling pups. Body weight (**A**), serum *p*PD oxidase activity (**B**), erythrocyte SOD1 activity (**C**), and hepatic copper concentrations (**D**) were assessed in suckling pups from dams fed diets varying in iron concentration for ~3.5 weeks. One-way ANOVA analysis results are as follows: Body weight (**A**) and *p*PD oxidase activity (**B**), *p* < 0.0001; erythrocyte SOD1 activity (**C**), *p* = 0.0002; hepatic Cu (**D**), *p* = 0.0051. When statistically significant differences were noted by one-way ANOVA, Tukey’s post hoc test was utilized to make comparisons between individual groups. Values without common superscript letters are statistically different from one another (*p* < 0.05) (**A**–**D**). All data points are shown, and the mean values are indicated by “+” signs. For hepatic Cu (**D**), 4 statistical outliers were detected (using the ROUT method, with Q set to 1%) and removed from the dataset.

The final body weights of the pups were highest in the 10X group and lower (by ~10%) in the 20× group (both as compared to the 1× group) (Figure 4A). Serum *p*PD oxidase activity trended lower in the 10× group and was significantly lower (by ~56%) in the 20× group (Figure 4B). Similarly, erythrocyte SOD1 activity in the pups showed a significant reduction (~42%) in the 20× group (Figure 4C). Additionally, hepatic copper was lower in the 10× and 20× groups (Figure 4D). Furthermore, unlike in experiment 1, hepatic iron content did not increase in the pups as dietary iron consumption increased in the dams (data not shown). This was an unexpected outcome but may be explained by the different experimental protocol that was utilized (i.e., the low-iron diet was started at a different time point during pregnancy and thus, the experimental diets (with variable iron content) were administered beginning at different times during pregnancy). Collectively, the data presented in Figure 4 demonstrate that high-iron consumption causes copper depletion in the suckling pups.

#### 3.1.5. Experiment 2: Increasing Dietary Iron to ~3-Fold above Requirements Decreases Intestinal Copper (^64^Cu) Absorption in Lactating Rat Dams

**Figure 5:**
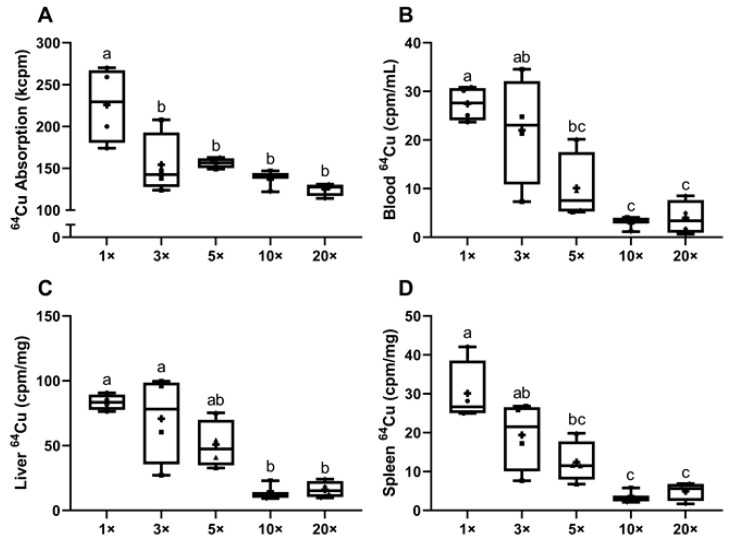
^64^Cu absorption and accumulation in blood, liver, and spleen were lower in iron-supplemented dams. Shown are intestinal copper (^64^Cu) absorption (**A**) and ^64^Cu activity in blood (**B**), liver (**C**), and spleen (**D**) of dams fed diets varying in iron concentration for ~3.5 weeks. One-way ANOVA analysis results are as follows: ^64^Cu absorption (**A**), *p* = 0.0019; ^64^Cu in blood (**B**) and liver ^64^Cu (**C**), *p* = 0.0004; and spleen ^64^Cu (**D**), *p* = 0.0002. When statistically significant differences were noted by one-way ANOVA, Tukey’s post hoc test was utilized to make comparisons between individual groups. Values without common superscript letters are statistically different from one another (*p* < 0.05) (**A**–**D**). All data points are shown, and the mean values are indicated by “+” signs.

Radiotracer ^64^Cu absorption studies were performed to test the hypothesis that impaired intestinal copper transport caused the noted copper deficiency in the dams fed high-iron diets. This is a logical postulate since iron and copper have similar physiochemical properties, and interactions between them are predictable and have indeed been extensively documented in the scientific literature [36]. Results showed that copper (^64^Cu) absorption (i.e., whole-body radioactivity (minus the GI tract)) was significantly decreased (~36%) in all dams consuming excess iron (as compared to the 1× group) (Figure 5A). Reflecting diminished intestinal copper transport, ^64^Cu in blood was lower in the 5× (~63% reduction), 10× (~90%) and 20× (~86%) groups (Figure 5B), while hepatic ^64^Cu accumulation was ~81% lower in the 10× and 20× groups (Figure 5C). Moreover, a positive correlation was noted between ^64^Cu absorption and liver ^64^Cu accumulation (*p* = 0.0017; *r* = 0.6711). Spleen ^64^Cu accumulation also showed a significant decrease in the 5× (~59% reduction), 10× (~88%) and 20× (~84%) groups (as compared to the 1× group) (Figure 5D). A significant correlation between ^64^Cu absorption and spleen ^64^Cu accumulation was also noted (*p* = 0.0006; *r* = 0.7135). These data suggest that high-iron consumption by lactating dams causes copper depletion due to impaired intestinal copper absorption.

#### 3.1.6. Experiment 2: Consumption of High-Iron Diets by Lactating Dams Decreases Copper Delivery to Suckling Pups

**Figure 6:**
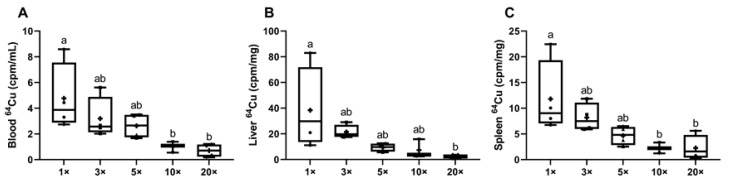
^64^Cu accumulation in blood, liver, and spleen were reduced in pups suckling dams that consumed diets with iron content in excess of requirements. ^64^Cu activity in blood (**A**), liver (**B**), and spleen (**C**) from pups at 13–15 days of age suckling dams that consumed diets varying in iron concentration for ~3.5 weeks. One-way ANOVA analysis results are as follows: blood ^64^Cu (**A**), *p* = 0.0153; liver ^64^Cu (**B**), *p* = 0.0357; and spleen ^64^Cu (C), *p* = 0.0174. When statistically significant differences were noted by one-way ANOVA, Tukey’s post hoc test was utilized to make comparisons between individual groups. Values without common superscript letters are statistically different from one another (*p* < 0.05) (**A**–**C**). All data points are shown (for 2 male and 2 female pups), and mean values are indicated by “+” signs.

Radiotracer ^64^Cu distribution in suckling pups was also assessed to investigate copper delivery through the dam’s milk. Blood and tissue ^64^Cu accumulation in the pups was consistent with the impaired intestinal copper absorption (and copper depletion) observed in lactating dams. Blood ^64^Cu content was lower in the 10× (~79%) and 20× (~85%) groups, as compared to the 1× group (Figure 6A). Liver ^64^Cu accumulation was also reduced, but only in the 20× group (~93% reduction) (Figure 6B). Blood ^64^Cu and liver ^64^Cu content data showed a strong correlation, as might be anticipated (*p* < 0.0001; *r* = 0.8489). Similarly, spleen ^64^Cu accumulation was lower by ~81% in the 10× and 20× groups (Figure 6C). A significant correlation was also noted between the blood ^64^Cu and spleen ^64^Cu content (*p* < 0.0001; *r* = 0.9482). Although we were unable to measure ^64^Cu in dam’s milk (for technical reasons), it is a logical postulate that copper delivery to the pups was lower since copper content in the milk was low. Copper depletion in the dams due to impaired intestinal copper absorption thus led to decreased copper delivery to suckling pups.

## 4. Discussion

The WHO recommends that pregnant women take 30–60 mg of supplemental iron daily, and for those who develop anemia, the recommendation is to consume an additional 60–90 mg of iron/day (for a total of 120 mg/day). Similar guidelines are provided by the Center for Disease Control for women in the U.S., with 30 mg/day of iron suggested for early pregnancy as a starting point and 60 mg/day upon the initial diagnosis of anemia (CDC Guidelines for Iron Supplementation During Pregnancy). When combined with iron naturally present in foods, iron added as a forticant to refined grain products and iron in daily vitamin/mineral supplements, total iron intake can be up to several times the established RDA for pregnancy. This is a potential concern as some individuals have a genetic propensity to absorb too much enteral iron (e.g., those with hereditary hemochromatosis) and excess (unabsorbed) iron can negatively affect the composition of the gut microbiota [37,38]. Further, as mentioned previously, high dietary iron could also induce copper deficiency, this increasing risk for abnormal pregnancy outcomes.

Here, our intent was to test the hypothesis that high intake of dietary iron in previously ID rat dams would have pathological outcomes for suckling pups. This investigation builds logically on our previous work in rats and mice demonstrating that iron intake at levels ≈5-fold above requirements causes copper depletion and associated pathological outcomes [13,14,15,16]. Although we are investigating the effect of high enteral iron on copper absorption, it is also important to consider possible influences of high iron on the absorption of other minerals (e.g., zinc). This issue has been researched and reviewed by others quite extensively. For example, Walker et al. summarized human iron and zinc supplementation trials and concluded that “zinc supplementation alone does not appear to have a clinically important negative effect on iron status” [39]. Moreover, Harvey et al. studied high-dose iron supplementation in pregnant women and noted that “no detectable adverse effects on zinc metabolism were observed after ingestion of 100 mg Fe/day” [40]. Whittaker, also studying the issue of iron/zinc interactions, found that iron intake at levels used in current food fortification programs has no adverse effects on zinc absorption [41]. Additionally, there is published evidence that iron supplementation (30 mg/day for 3 months) does not cause zinc deficiency in healthy one-year-old infants [42]. Therefore, based upon precedence established in the scientific literature, high iron intake is unlikely to impair zinc absorption.

In the current investigation, in experiment 1, as dietary iron increased, copper depletion became apparent in suckling pups as exemplified by decreases in serum Cp activity (i.e., *p*PD oxidase activity), and concomitant increases in hepatic nonheme iron. In experiment 2, which had an optimized design, copper depletion was noted in dams and pups. For example, in lactating dams, as dietary iron increased, serum Cp activity and liver copper content decreased, and hepatic nonheme iron increased. In pups, when dams consumed diets with iron above requirements, plasma Cp and serum SOD1 activities were decreased, and liver copper was depleted. High dietary iron thus precipitates the development of copper deficiency in lactating dams and their suckling pups.

Changes in body copper status could reflect changes in the assimilation of dietary and supplemental copper, or alternatively, hepatic copper excretion in bile could be altered. Modulation of enteral copper transport and biliary copper excretion are both thought to contribute to whole-body copper homeostasis [43]. We hypothesized that high-iron intake antagonized intestinal copper absorption, which would be consistent with previous studies supporting this possibility [11]. Therefore, copper (^64^Cu) absorption studies were undertaken as part of the current investigation. Supporting this postulate, ^64^Cu absorption and accumulation in various tissues was lower in lactating dams consuming excess dietary iron. A similar trend was noted in suckling pups, that is, ^64^Cu accumulation in all tissues assayed was progressively lower as iron intake in the dams increased. There are thus two (possible) logical conclusions: (1) high enteral iron inhibits absorption of dietary copper in the dams causing low blood copper and low copper transfer across the placenta during fetal development; and (2) high dietary iron blocks intestinal copper transport in the dams during lactation leading to low blood and milk copper content, thus decreasing copper delivery to the suckling pups. It would seem likely then that both scenarios would contribute to copper depletion in offspring, but definitive proof awaits future experimentation. In this investigation, we attempted to obtain milk from lactating dams after oral gavage with the ^64^Cu transport solution, but due to technical issues, were unable to do so.

One final issue to consider relates to the mechanism by which iron supplementation causes copper depletion. Copper transporter 1 (Ctr1) is the main intestinal copper transporter (at least in mice) [44], while the main iron transporter (in rodents and probably humans) is divalent metal-ion transporter 1 (Dmt1) [45]. Interestingly, Dmt1 has been suggested to also transport copper [46,47,48], but to our knowledge, there is no evidence that Ctr1 can transport or otherwise interact with enteral iron. Logically, excess enteral iron could block copper transport by Ctr1, possibly by competing for copper binding to the pore region of the Ctr1 heterotrimer. An additional possibility relates to the intestinal reductase enzyme which reduces dietary cupric copper (to cuprous copper, which is then imported by Ctr1). This reductase could also reduce dietary iron. For example, the cytochrome b reductase 1 (a ferrireductase) has also been suggested to reduce dietary copper [49]. If this (or another) reductase can act upon dietary iron and copper, then this could be another step by which excess iron could impair copper absorption. Moreover, if Dmt1 was responsible for some proportion of copper transport, excess iron could out compete copper for binding to the extracellular domain of the Dmt1 protein. Another possibility relates to whether high-iron intake by the dam could inhibit placental copper delivery to the developing fetuses. Placental iron and copper transfer from the maternal circulation to the fetuses occur by distinct mechanisms. Iron transfer involves transferrin receptor 1 (Tfr1) (iron import) and ferroportin 1 (Fpn1) (iron export) [50,51]. Copper is transported across the placenta by Ctr1 (copper import) and the copper-transporting ATPase, Atp7a (copper export) [52]. Given notable iron/copper interactions in other tissues (e.g., intestine and liver), the possibility exists that high blood iron could inhibit copper transport into placental tissue by Ctr1 (as may also occur in the small intestine with high enteral iron). However, it seems unlikely that iron intake at the levels used in this investigation would sufficiently increase blood iron content to have such inhibitory effects on placental copper transport. In sum, future experimentation will be required to elucidate the mechanism(s) by which iron supplementation suppresses intestinal and/or placental copper transport.

## 5. Conclusions

This investigation, which utilized a rat model of ID/iron supplementation during pregnancy and lactation, has demonstrated that dietary iron in excess of requirements impairs copper absorption and causes copper deficiency in lactating dams and their suckling pups. These findings support the notion that iron supplementation, which is widely recommended in pregnant women due to the high incidence of ID and anemia, should be undertaken with caution [9]. Excess enteral iron may inhibit intestinal copper transport, as demonstrated here, but it can also cause GI tract irritation and mucosal damage, and constipation, and it may also alter the composition of the gut microbiota towards a more pathogenic state [53]. Thus, iron supplements should be taken at the lowest effective dose (which may need to be empirically determined). One simple solution that would obviate the issue with copper absorption would be to include extra copper in iron supplements (as has been previously suggested) [11]. This seems reasonable especially since the UL for copper of 10,000 µg/day is 10-fold higher than the RDA for pregnant women (i.e., 1000 µg/day) and >7-fold higher than the RDA for lactating women (i.e., 1300 µg/day), so there is a large margin for error.

## Figures and Tables

**Table 1:** Common ingredients in all 5 experimental diets.

Ingredient	Amount (g/kg)
Cornstarch Casein Dyetrose	397 200 132
Sucrose ^1^	100
Soybean oil	70
Cellulose (micro)	50
Mineral Mix ^2^	35
Vitamin Mix ^3^	10
L-Cystine Choline Bitartrate t-Butylhydroquinone	3 2.5 0.014

^1^ Small amounts of sucrose were replaced with carbonyl iron in the 5×, 10×, and 20× diets; ^2^ AIN-93-GX standard mineral mixture lacking iron and copper; ^3^ AIN-93-VX standard complete vitamin mixture.

**Table 2:** Iron and copper composition and content, and energy density of experimental diets.

Ingredient	1× Diet	3× Diet	5× Diet	10× Diet	20× Diet
Ferric citrate ^1^	80	80	80	80	80
Carbonyl Fe ^1^	0	160	320	720	1520
Cupric carbonate ^1^	2.5	2.5	2.5	2.5	2.5
**Mineral & Energy Content**					
Iron ^1,2^	88	235	422	886	1598
Copper ^1,2^	2.79	2.75	2.44	3.01	2.18
Energy (kcal/kg)	3760	3760	3759	3757	3754

^1^ Values are in mg/kg (or ppm); ^2^ Determined by inductively coupled plasma mass spectrometry (ICP-MS) analysis of 10 randomly selected diet pellets.

**Table 3:** Experimental outcomes.

Experiment 1: Dam ^1^						
Parameter	*p* ^2^	1× Fe	3× Fe	5× Fe	10× Fe	20× Fe
Hb (g/dL)	ns	12.8 ± 0.9	12.6 ± 1.0	13.9 ± 0.8	11.8 ± 1.1	10.3 ± 1.8
Serum NH Fe (µg/dL)	ns	429.7 ± 41.3	331.2 ± 67.9	414.2 ± 64.2	263.6 ± 103.8	358.3 ± 52.2
TSAT (%)	ns	65.3 ± 8.8	72.0 ± 18.5	92.7 ± 14.1	59.6 ± 25.0	77.4 ± 8.3
*p*PD oxidase activity	ns	133.3 ± 89.2	67.2 ± 1.4	48.6 ± 22.4	137.5 ± 94.8	69.3 ± 2.2
TIBC (µg/dL)	< 0.0001	660.6 ± 28.8 ^a^	466.1 ± 43.1 ^b^	446.8 ± 1.3 ^b^	448.9 ± 31.3 ^b^	461.9 ± 25.9 ^b^

^1^ Data are means ± SD; *n* = 2–4 rats/group; ^2^ Analyzed by one-way ANOVA. Since TIBC analysis showed significance, these data were subjected to post hoc analysis. Values without common superscript letters are statistically different from one another (*p* < 0.05).; Hb (hemoglobin); NH (nonheme); *p*PD (*para*-phenylenediamine); TSAT (transferrin saturation); TIBC (total iron-binding capacity); ns (not significant).

**Table 4:** Organ weights in experiment 1.

Dams ^1^		Suckling Pups ^2^			
Organ	% of BW ^3^	Organ	% of BW ^3^	Fe (*p*) ^4^	Sex (*p*) ^4^
Liver	5.46 ± 0.47	Liver	3.24 ± 0.31	*p* < 0.0001	*p* = 0.0237
Kidney	0.69 ± 0.04	Kidney	1.00 ± 0.09	*p* = 0.0201	*p* = 0.0225
Spleen	0.26 ± 0.03	Spleen	0.46 ± 0.08	*p* = 0.0005	ns
Heart	0.42 ± 0.02	Heart	0.57 ± 0.07	*p* < 0.0001	ns

^1^*n* = 15 for liver, kidney, spleen, and heart; ^2^ *n* = 20–38 (males and females) for liver, kidney, spleen, and heart; ^3^ Data are means ± SD; ^4^ Analyzed by one-way ANOVA; BW (body weight); ns (not significant).

**Table 5:** Dam’s organ weights in experiment 2.

Organ ^1^	% of BW ^2^	Liver % of BW ^1–3^	Diet
Kidney	0.73 ± 0.06	4.77 ± 0.41 ^b^	1×
Spleen	0.23 ± 0.03	4.63 ± 0.41 ^b^	3×
Heart	0.37 ± 0.03	5.64 ± 0.57 ^ab^	5×
		5.77 ± 0.18 ^ab^	10×
		5.96 ± 0.70 ^a^	20×

^1^*n* = 3–4/group; ^2^ Data are means ± SD; ^3^ Analyzed by one-way ANOVA, *p* = 0.006. Values without common superscript letters are statistically different from one another (*p* < 0.05).

**Table 6:** Pup’s organ weights in experiment 2.

Organ ^1^	% of BW ^2^	Heart % of BW ^1−3^	Diet
Liver	3.28 ± 0.29	0.62 ± 0.04 ^ab^	1×
Kidney	1.28 ± 0.11	0.56 ± 0.07 ^b^	3×
Spleen	0.48 ± 0.08	0.56 ± 0.08 ^b^	5×
		0.61± 0.07 ^ab^	10×
		0.72 ± 0.09 ^a^	20×

^1^*n* = 6–8/group; ^2^ Data are means ± SD; ^3^ Analyzed by one-way ANOVA, *p* = 0.0004. Values without common superscript letters are statistically different from one another (*p* < 0.05).

## Data Availability

Not applicable.
